# Sexual and Gender Identity-Based Microaggressions: Differences by Sexual and Gender Identity, and Sex Assigned at Birth Among Dutch Youth

**DOI:** 10.1177/08862605211056729

**Published:** 2021-12-04

**Authors:** Wouter J. Kiekens, Tessa M. L. Kaufman, Laura Baams

**Affiliations:** 1 Department of Sociology/Interuniversity Center for Social Science Theory and Methodology (ICS), 403520 University of Groningen, The Netherlands; 2 Department of Education and Pedagogy, 8125 Utrecht University, The Netherlands; 3 Department of Pedagogy and Educational Sciences, 3647 University of Groningen, The Netherlands

**Keywords:** aexual and gender minority youth, icroaggressions, sexual identity, gender identity, sex at birth

## Abstract

Research describes several sexual and gender identity-based microaggressions that sexual and gender minority (SGM) people might experience. We aimed to examine the occurrence of different sexual and gender identity-based microaggressions among SGM youth and to identify differences by sexual and gender identity, and sex assigned at birth. Open-ended questions about daily experiences were coded for 16 types of sexual and gender identity-based microaggressions in two daily diary studies among Dutch SGM youth (Study 1: *N* = 90, *M* age = 17.64 *SD* = 1.78; Study 2: *N* = 393, *M* age = 18.36 *SD* = 2.65). Several types of microaggressions were identified, and there was sizable variability in the reported frequency. Overall, lesbian women and bisexual youth were less likely to report microaggressions than gay youth. Bisexual youth were less likely to report use of heterosexist or transphobic terminology than gay youth and youth assigned male at birth were less likely to report invalidation of LGBTQ identity than youth assigned female at birth. Last, gender minority youth were more likely to report familial microaggressions, invalidation of LGBTQ identity, and threatening behaviors than cisgender youth. Overall, this study provides empirical support using mixed qualitative and quantitative methods for theorized typologies of microaggressions among Dutch SGM youth.

Sexual and gender minority (SGM) youth face stigma related to their sexual and gender identity (Meyer, 2003; Testa et al., 2015). In their day-to-day lives, this stigma can manifest in the form of subtle mistreatments, also referred to as sexual and gender identity-based microaggressions. Microaggressions, originally studied among racial/ethnic minority groups (Pierce et al., 1977), are understood as “brief and commonplace daily verbal, behavioral, or environmental indignities, whether intentional or unintentional, that communicate hostile, derogatory, or negative slights and insults” (Sue et al., 2007, 273). Studies have described microaggressions that may be unique to SGM people, referred to as sexual and gender identity-based microaggressions, and shows that these microaggressions are associated with poorer mental health (Kaufman et al., 2017; Lui & Quezada, 2019). However, it is unclear what types of microaggressions SGM youth experience and whether some SGM subgroups are at risk of experiencing different types of microaggressions. An overview of commonly experienced microaggressions and the occurrence of microaggressions in a larger quantitative framework will improve our understanding of adverse experiences among SGM youth and enable us to tailor prevention and intervention efforts. With this study, we aimed to identify to what extent SGM youth experienced previously described types of microaggressions. Further, we aimed to investigate differences by sexual and gender identity, and sex assigned at birth in the occurrence of microaggressions.

## Sexual and Gender Identity-Based Microaggressions

Microaggressions can take the form of microassaults, microinsults, and microinvalidations (Sue et al., 2007). Microassaults are described as conscious attitudes or beliefs communicated to marginalized groups through environmental cues, verbalizations, or behaviors. These messages can be subtle or explicit and are closely related to traditional discrimination. In contrast, microinsults are interpersonal or environmental messages that are unintentional, but they convey stereotypes, rudeness, and insensitivity. Last, microinvalidations are understood as messages or environmental cues that invalidate the experiences of a marginalized group. These messages are considered most covert and insidious as they directly invalidate people’s experiences (Sue et al., 2007).

Regardless of the form of microaggressions, several types of sexual gender identity-based microaggressions have been proposed (Nadal et al., 2010; Sue, 2010). Initially, Sue (2010) described a list of sexual and gender identity-based microaggressions, and Nadal and colleagues (2010) proposed a more comprehensive list of sexual and gender identity-based microaggression types (see Table 1). Empirical qualitative research has shown the occurrence of some of these sexual and gender identity-based microaggression types and identified additional types as well. For example, a surface level of acceptance only when one is not involved in a relationship (*undersexualization*, Platt & Lenzen, 2013) and hurtful jokes (*microaggression as humor*, Platt & Lenzen, 2013). Further, additional types of microaggressions include non-physical assaultive experiences (*threatening behaviors*, Nadal et al., 2011), an ever-present threat of verbal harassment or physical violence (*physical threat or harassment*, Nadal et al., 2012), entitlement by others to objectify one’s body (*denial of body privacy*, Nadal et al., 2012), disapproval by family in a microaggressive manner (*familial microaggressions*, Nadal et al., 2012), the presence of environmental or systematic microaggressions (*systematic microaggressions*, Nadal et al., 2012), and the questioning or undermining of one’s sexual or gender identity by others (*invalidation of *lesbian, gay, bisexual, transgender, and queer [LGBTQ]* identity*, Munro et al., 2019).

**Table 1.** Description and Example of Sexual and Gender Identity-Based Microaggression Types, Number and Percentage of Participants that Reported a Microaggression, and the Total Number a Microaggression was Reported.

Sexual and Gender Identity-Based Microaggression Types	Description	Example from Data	Study 1^a^ (*N* = 90)		Study 1^a^ (*N* = 90)	
			*N* (%) Participants Reporting a Microaggression	*T*imes Microaggression was Reported	*N* (%) Participants Reporting a Microaggression	*T*imes Microaggression was Reported
Discomfort/disapproval of the LGBTQ experience (Nadal et al., 2010)	The treatment of SGM people with disrespect or condemnation	Last Friday, a teacher said that he hates lesbians.	22 (24)	24	65 (17)	84
Use of heterosexist or transphobic terminology (Nadal et al., 2010)	Use of heterosexist language to degrade SGM people	Someone I didn’t know called something “gay” at a party.	48 (53)	75	45 (12)	61
Invalidation of LGBTQ identity (Munro et al., 2019)	The undermining and questioning of SGM identities	At school, people said that being gay is fake, and people only identify as gay to get attention.	10 (11)	11	32 (8)	40
Microaggressions as humor (Platt & Lenzen, 2013)	The communication of a microaggression as a joke	Hearing the word “gay” being used as a joke.	19 (21)	27	29 (7)	42
Threatening behaviors (Nadal et al., 2011)	Verbal harassment	I was called names on the street 4 times.	(0)	0	25 (6)	35
Familial microaggressions (Nadal et al., 2012)	Disapproval of one’s (gender) identity by family	As a girl, I have a boyfriend. We have a healthy relationship, but my parents do not believe that you can be attracted to both sexes.	13 (14)	14	25 (6)	30
Endorsement of heteronormative or gender-conforming culture/behaviors (Nadal et al., 2010)	The expectation that SGM people “act straight/cisgender”	Someone said “Act more feminine!” to me.	20 (22)	20	15 (4)	18
Assumption of sexual pathology/abnormality (Nadal et al., 2010)	The over-sexualization of SGM people and the presumption they are sexual deviants	The boyfriend of my best friend (female) said I should hang out with her less because I am a lesbian. He said I could pose a threat to him	11 (12)	11	12 (3)	13
Assumption of universal LGBTQ experience (Nadal et al., 2010)	The assumption that all SGM people are the same/have the same experiences	Someone said “but you are a girly-girl, lesbians and bisexuals are usually masculine.”.	25 (28)	31	10 (3)	10

Exoticization (Nadal et al., 2010)	The fetishization and dehumanization of SGM people	I was chatting with a guy online, and when he figured out I was pansexual, he immediately asked if I was down for a threesome.	5 (6)	6	10 (3)	10
Systemic and environmental microaggressions (Nadal et al., 2010)	Institutional or communal (transphobic) microaggressions	During a church service, they said that if you are attracted to the same sex, you are a sinner.	3 (3)	3	8 (2)	8
Denial of personal body privacy (Nadal et al., 2012)	The objectification of SGM people’s body	My employer questioned me extensively about what kind of surgery I wanted to get when I transition.	3 (3)	4	6 (2)	7
Denial of individual heterosexism/transphobia (Nadal et al., 2010)	Denial of individual heterosexist bias or prejudice	Being addressed as “lady” because of my sexual orientation. I was accused of being sensitive when I confronted them about this.	2 (2)	2	1 (0)	1
Undersexualization (Platt & Lenzen, 2013)	Acceptance of sexual minority identity if a person is not in a relationship	I wanted to sleepover on a date, which made my mother very uncomfortable, and I know she is still not okay with my sexuality.	(0)	0	1 (0)	1

Physical threat or harassment (Nadal et al., 2012)	The presence of (the threat of) verbal harassment, physical violence, and the ever-present threat of such violence	I felt unsafe and intimidated on the train because a group of guys moved closer to me, stared at me, and laughed.	2 (2)	2	1 (0)	1
Denial of the reality of heterosexism/transphobia (Nadal et al., 2010)	The denial of the existence of heterosexist/homophobic experiences	Today a guy made the remark that he is ok with me being gay but that I should not try to fight against homophobia in school because he thought it was useless.	2 (2)	2	0 (0)	0

^a^Microaggression types are ordered based on the number of participants that reported a microaggression in Study 2.

Taken together, empirical research has shown the occurrence of these types of sexual and gender identity-based microaggressions and identified additional ones (Munro et al., 2019; Nadal et al., 2011, 2012; Platt & Lenzen, 2013). However, research into the occurrence of all these microaggressions is limited, especially with regard to the additional types of microaggressions. By not examining these microaggressions in their entirety, a comprehensive understanding of SGM youth’s experiences is obstructed.

Studying the occurrence of sexual and gender identity-based microaggressions among SGM youth is especially relevant because this is the age period during which sexual and gender identity develop (Bilodeau & Renn, 2005). For SGM youth, it is therefore important to, among others, overcome internalized stigmatic messages (Bilodeau & Renn, 2005). Considering that microaggressions communicate daily hostile, derogatory, or negative slights and insults (Sue et al., 2007), they can negatively affect identity development among SGM youth and ultimately their health (Mallory et al., 2021).

## Subgroup Differences

Sexual and gender minority youth’s experiences with microaggressions are suggested to be heterogenous (Nadal et al., 2016). This heterogeneity might be rooted in youth's unique experiences with a specific sexual or gender identity (Meyer, 2003; Testa et al., 2015). For example, bisexual people may experience different types of microaggressions than lesbian and gay people (Sarno & Wright, 2013), which may stem from biphobia from heterosexual *and* sexual minority communities (Huffaker & Kwon, 2016). There may also be differences by sex assigned at birth in the experience of microaggressions. This can manifest in daily indignities that cisgender women experience more often than cisgender men (Lewis, 2017; Swim et al., 2001), leading to sex-based differences in the occurrence of microaggressions (Nadal et al., 2011). Also, the level of acceptance of minority groups may lead to specific types of microaggressions. For example, among SGM groups, transgender people experience the highest levels of prejudice and stigma compared to cisgender sexual minority people (Martín-Castillo et al., 2020; Su et al., 2016). The differences in acceptance could result in different degrees of severity and different types of microaggressions. Despite some preliminary studies that explored group-based differences (Nadal et al., 2016), there is currently little research examining a comprehensive set of microaggressions and group-based differences therein.

## The Present Study

The present study has two aims. First, to examine whether SGM youth report sexual and gender identity-based microaggressions that were identified in previous studies (Munro et al., 2019; Nadal et al., 2010, 2011, 2012; Platt & Lenzen, 2013). Second, to investigate differences in the experience of microaggressions by sexual and gender identity, and sex assigned at birth. We utilized data from two samples of Dutch SGM youth. Data from the first study were used to examine which sexual and gender identity-based microaggressions SGM youth reported and in the second study, we were additionally able to study differences by sexual and gender identity, and sex assigned at birth.

## Study 1

### Method

#### Procedure

Data came from a larger research project on the occurrence and correlates of sexual and gender identity-based microaggression experiences among Dutch sexual minority youth (Baams et al., 2018; Kaufman et al., 2017). Participants were recruited through advertisements on websites and social media from several LGBT community-based organizations throughout the Netherlands in 2015. Participants were asked to complete an online survey and were entered into a raffle to receive a €5 gift card for their participation. After completing a baseline questionnaire, participants were asked to participate in an 8-day daily diary study. They were informed that their participation was confidential and voluntary. Informed consent was obtained from all participants included in the study. Participants had the opportunity to participate anonymously, end the questionnaire at any time, and skip any question. Approval of all procedures was granted by the Ethics Committee of the Social and Behavioural Sciences Faculty at Utrecht University.

Using data from a daily diary study has the advantage that participants reflect on their experiences more momentarily. That is, in most surveys participants are asked about their *past* experiences with, for instance, microaggressions. However, people might have difficulties reconstructing these experiences (Larson & Csikszentmihalyi, 2014), especially when these are related to discrimination (Sechrist et al., 1998). Using diary data overcomes this shortcoming because youth are asked about their experiences on a daily basis.

### Participants

In total, *N* = 364 youth participated in the research project. For this study, data from participants were excluded if they were not between 16 and 22 years old (*n* = 94), had a missing value on sexual identity, sex assigned at birth, gender identity, identified as cisgender *and* heterosexual (*n* = 65), and if they did not participate in the 8-day daily diary study (*n* = 115), resulting in a final analytic sample of *N* = 90.

The sample was diverse concerning sexual identity, with 33 (37%) participants identifying as lesbian, 22 (24%) as gay, 28 (31%) as bisexual, 2 (2%) as queer, and 5 (6%) with another sexual minority identity. In total, 66 (73%) were assigned female and 24 (27%) were assigned male at birth. Concerning gender identity, 80 (89%) identified as cisgender and 10 (11%) as a gender minority (i.e., participants gender identity was not concordant with their sex assigned at birth). Mean age was 17.64 (*SD* = 1.78), 70 (78%) lived with their parents, and 8 (9%) participants did not identify as Dutch but had a migration background from, for instance, Morocco or Turkey. Last, 46 (51%) were in high school, 10 (11%) in vocational education, 25 (28%) attended (applied) university, and 3 (3%) did not attend school anymore at the time of the study.

#### Measures

##### Microaggressions

Participants were presented with an open-ended question that was developed for this study together with experts in the field “Many people experience situations in which something is said about one’s sexual orientation. For example, a joke, an unexpected comment, or the use of a certain word. These remarks can, but do not have to, have bad intentions. We wonder if you had such an experience today, what happened, how you felt, and how you responded. We would like to hear anything you want to share! Sometimes these experiences cause feelings of shame, anger, or sadness. And sometimes these remarks do not really stand out, but they still leave you with an uneasy feeling.” If participants did not have such an experience, they could skip this question. On the first day of the daily diary study, participants could also describe experiences in the past year. Two coders independently coded the answers to this open-ended question for 16 types of sexual and gender identity-based microaggressions that were identified from the literature (see Table 1). Thus, we took a deductive approach to data coding: codes were theoretically driven and a codebook was created prior to analyzing the data (Crabtree & Miller, 1999). After coding the open-ended questions, the two coders compared their codes. When different codes were used, coders would discuss until consensus was reached. One diary entry could be coded as multiple types of microaggressions. Some entries to the open-ended questions did not describe microaggressions, and others were not described clearly enough to code as a microaggression type. Neither of these entries were included in the analyses (*n* = 48).

#### Analytic Strategy

Because of the small sample size, we only assessed how many participants reported a specific sexual and gender identity-based microaggression type across all days of the study to validate existing microaggression types described in the literature.

### Results

Table 1 presents the number and percentage of participants who reported a sexual and gender identity-based microaggression and the frequency a microaggression was reported by all participants. The mean number of days that participants completed a diary entry was 4.64 (*SD* = 2.34) out of 8 days. In total, 79 (88%) of all participants reported at least one sexual and gender identity-based microaggression. The mean number of microaggressions reported during the study was 2.58 (*SD* = 2.09).

Most microaggression types were reported by at least one participant. The three microaggression types that were reported by most participants described experiences where heterosexist/transphobic language was used to degrade participants (*use of heterosexist or transphobic terminology*, reported by 53% of all participants), experiences in which it was assumed that all SGM people are similar/have similar experiences *(assumption of universal LGBTQ experience,* reported by 28% of all participants), and experiences in which participants felt that they were treated with disrespect or condemnation (*discomfort/disapproval of the LGBTQ experience,* reported by 24% of all participants). None of the participants reported experiences of a surface level of acceptance only when one is not involved in a relationship (*undersexualization)* or non-physical assault (*threatening behaviors)*.

### Conclusion

Almost all sexual and gender identity-based microaggression types were reported in an 8-day period. More than half of SGM youth experienced *heterosexist or transphobic terminology*. In contrast, none of the participants experienced *undersexualization* or *threatening behaviors*. Thus, SGM youth in this sample reported most of the sexual and gender identity-based microaggressions described by previous studies, but there was large variability in how many participants reported specific microaggression types.

## Study 2

In Study 1, we were only able to examine what sexual and gender identity-based microaggressions SGM youth reported in a relatively small sample of SGM youth. In Study 2, we examine what sexual and gender identity-based microaggressions SGM youth report in a larger sample, which strengthens our findings. Additionally, the larger sample additionally makes it possible to explore differences by sexual and gender identity, and sex assigned at birth.

### Method

#### Procedure

Data came from a 14-day daily diary study. Participants were recruited through Facebook and Instagram advertisements that ran from October to December 2019. Advertisements were targeted at 16–25-year-old youth that lived in the Netherlands, spoke Dutch, and had sexual and gender identity-related interests (i.e., Gay Pride Parade). For each completed daily survey, participants received approximately €1.79, which could amount to €25 in gift cards. Participants were informed that their participation was confidential and voluntary, and all participants provided informed consent. On the first day of the study, participants completed a baseline survey and immediately after that the first daily diary survey. The Ethics Committee of the the Pedagogy and Educational Sciences Department at the University of Groningen approved the study’s procedure.

#### Participants

The sample comprised of *N* = 409 participants. Data from participants who completed the consent form but did not complete a question (*n* = 13) or a daily survey (*n* = 3) were excluded, resulting in a sample of *N* = 393. There was no missing data for sexual or gender identity, and sex assigned at birth, and no participants identified as cisgender *and* heterosexual. The sample was diverse in terms of sexual identity, with 65 (17%) of participants identifying as lesbian, 107 (27%) as gay, 116 (30%) as bisexual, 29 (7%) as queer, 39 (10%) as pansexual, 5 (1%) as heterosexual, 17 (4%) did not know their sexual identity, and 15 (4%) had a different minority sexual identity (write-in options). For sex assigned at birth, 252 (64%) were assigned female and 141 (36%) were assigned male at birth. In total, 127 (32%) identified as cisgender men, 182 (46%) as cisgender women, 22 (6)% as transgender men, 3 (1%) as transgender women, 15 (4%) as non-binary, 8 (2%) as genderqueer, 4 (1%) as genderfluid, 3 (1)% had a different gender identity, and 29 (7)% did not know their gender identity. Mean age was 18.36 (*SD* = 2.65) and 296 (75%) lived with their parents. Of all participants, 53 (13%) had a migration background from, for instance, Morocco, Surname, or the Antilles. Last, 130 (33%) were in high school, 75 (19%) in vocational education, 107 (27%) attended (applied) university, and 76 (19%) did not attend school anymore at the time of the study.

#### Measures

##### Microaggressions

Experiences with sexual and gender identity-based microaggressions were assessed by asking participants, “Since filling in the previous daily survey (on the first day: ‘In the past 24 hours’), did you have a negative experience related to your sexual orientation or gender identity? For example, annoying jokes, inappropriate questions, being excluded, or being called names.” This item was adapted from a daily diary study on heterosexism (Mohr & Sarno, 2016). Answer categories were 0 = *No* and 1 = *Yes*. When *Yes* was selected, participants were asked “Could you describe this negative experience? You can be as elaborate as you want.” Similar to Study 1, the answers to these open-ended questions were independently coded by two coders for 16 types of sexual and gender identity-based microaggressions (see Table 1). Thus, we took a deductive approach to coding the data (Crabtree & Miller, 1999). After coding, the two coders compared their codes. Coders would discuss until consensus was reached. One diary entry could be coded as multiple types of microaggressions. Some entries to the open-ended questions did not describe microaggressions, and others were not described clearly enough to code as a microaggression type. Neither of these entries were included in the analyses (*n* = 50). Besides all the different microaggression types, we also created a variable indicating if a participant reported at least one microaggression type during the study named *Any microaggression*.

##### Sexual Identity

Sexual identity was assessed with the item “How would you describe your sexual identity?” with answer options: 1 = *Lesbian*, 2 = *Gay*, 3 = *Bisexual*, 4 = *Queer*, 5 = *Pansexual*, 6 = *Heterosexual*, 7 = *I don’t know*, and 8 = *Other, namely*. Two female participants identified as gay. Because these participants used an identity that indicated same-sex attraction as cisgender women, we refer to them as *Lesbian* throughout the manuscript*.* Sexual identity was recoded as 0 = *Gay*, 1 = *Lesbian*, 2 = *Bisexual,* and 3 = *Queer/Pansexual/Heterosexual/I don’t know/Other, namely.*

##### Sex assigned at birth and gender identity

To assess sex assigned at birth and gender identity we used the recommended two-step approach (The GenIUSS Group, 2014). Sex assigned at birth was assessed with the following item: “What is the sex you were assigned at birth?” with answer options 1 = *Male*, 2 = *Female*, and 3 = *Other, namely*. There were no participants that answered 3 = *Other.* Responses were recoded such that 0 = *Female* and 1 = *Male*. Gender identity was assessed with the following item “How would you describe your gender identity?” with answer options 1 = *Man*, 2 = *Woman*, 3 = *Transgender man*, 4 = *Transgender woman***,** 5 = *Non-binary*, 6 = *Genderqueer*, 7 = *Genderfluid*, 8 = *Don’t know*, and 9 = *Other, namely*. Because of a relatively small number of participants that identified as a gender minority, we recoded this as 0 = *Cisgender* if participants had concordant sex assigned at birth and gender identities (*Female* or *Male* assigned at birth, and *Woman* or *Man* as gender identity, respectively). If participants reported a gender identity that was not concordant with their sex assigned at birth, they were considered 1 = *Gender minority*.

##### Number of days of participation

A variable was created that reflected the number of days participated in the daily diary study.

#### Analytic Strategy

Similar to Study 1, we assessed reports of sexual and gender identity-based microaggression types across all study days. Second, to study group-based differences in microaggressions, separate analyses were conducted with each sexual and gender identity-based microaggression type as the dependent variable. In all these analyses, sexual identity, gender identity, and sex assigned at birth were simultaneously included as independent variables. In some cases, the dependent variable was almost perfectly predicted by the independent variables, which means that for specific values of the independent variable the dependent variable would almost always take the same value. This is also referred to as quasi-separation and leads to inflated estimates. To handle this, we conducted Firth logistic regression analyses, which use a penalized likelihood estimation method (Firth, 1993; Leitgöb, 2020).

Further, the rule of thumb for logistic regression analyses is to have 10 outcome events per independent variable. However, simulation studies have shown that this can be relaxed to 5–9 events per independent variable (Vittinghoff & McCulloch, 2007). Therefore, we conducted analyses only when a specific microaggression type was reported at least 20 times during the study (4 independent variables × 5 events = a minimum of 20 events per analysis). Because of this, analyses of subgroup differences were only conducted for the following microaggression types: *Use of heterosexist or transphobic terminology, discomfort/disapproval of the LGBTQ experience, microaggressions as humor, familial microaggressions, invalidation of LGBTQ identity,* and *threatening behaviors*. All analyses were performed in Stata 16 (StataCorp, 2019) and we controlled for number of days of participation in the Firth logistic regression analyses.

### Results

Table 1 presents the number and percentage of participants who reported a sexual and gender identity-based microaggression and the frequency a microaggression was reported by all participants. The mean number of days that participants completed a diary entry was 12.02 (*SD* = 3.51) of 14 diary days. In total, 155 (39%) of all participants reported at least one sexual and gender identity-based microaggression. The mean number microaggressions reported during the study was 0.92 (*SD* = 1.63).

The three microaggression types that were reported by most participants described experiences in which participants felt that they were treated with disrespect or condemnation (*discomfort/disapproval of the LGBTQ experience*, reported by 17% of all participants), experiences in which heterosexist/transphobic language was used to degrade participants (*use of heterosexist or transphobic terminology*, reported by 12% of all participants), and experiences in which participant’s SGM identity was undermined or questioned (*invalidation of LGBTQ* *identity,* reported by 8% of all participants). Only experiences in which the existence of heterosexist/homophobic was denied (*denial of the reality of heterosexism/transphobia)* were not reported by participants.

Table 2 presents the results of the Firth logistic regression analyses in which sexual identity, gender identity, and sex assigned at birth were used to predict the occurrence of microaggression types. Lesbian (*OR* = .26, 95% *CI*: .09–.71) and bisexual (*OR* = .38, 95% *CI*: .16–.91) participants had lower odds of reporting any microaggression type than gay participants. Focusing on specific microaggression types, bisexual participants had lower odds of reporting experiences in which heterosexist/transphobic language was used (*use of heterosexist or transphobic terminology, OR* = .21, 95% *CI:* .04–.99) than gay participants. Participants assigned male at birth had lower odds of reporting experiences in which their SGM identity was undermined or questioned (*invalidation of LGBTQ identity*, *OR* = .004, 95% *CI*: .0001–.35) than participants assigned female at birth. Last, gender minority participants had higher odds of reporting experiences in which others questioned their sexual or gender identity (*invalidation of LGBTQ identity, OR* = 2.74, 95% *CI*: 1.19–6.32), higher odds of reporting disapproval by family in a microaggressive manner (*familial microaggressions, OR* = 4.46, 95% *CI*: 1.71–11.63), and higher odds of non-physical assault (*threatening behaviors, OR* = 4.58, 95% *CI*: 1.65–12.71) than cisgender participants.

**Table 2.** Firth Logistic Regression Analyses with Sexual Identity, Gender Identity, and Sex Assigned at Birth Predicting Microaggression Types for Study 2

	Sexual Identity (ref = Gay)						Sex Assigned at Birth (ref = Female)		Gender Identity (ref = Cisgender)	
	Lesbian		Bisexual		Other		Male		Gender minority	
	*OR*	95% *CI*	*OR*	95% *CI*	*OR*	95% *CI*	*OR*	95% *CI*	*OR*	95% *CI*
Any microaggression	**.26**	[.09, 0.71]	**.38**	[.16, 0.91]	.56	[.23, 1.35]	.55	[.26, 1.21]	1.55	[.90, 2.69]
Discomfort/disapproval of the LGBTQ experience	.44	[.11, 1.71]	.49	[.15, 1.65]	.64	[.19, 2.14]	.53	[.18, 1.57]	1.56	[.79, 3.08]
Use of heterosexist or transphobic terminology	.24	[.04, 1.32]	**.21**	[.04, 0.99]	.24	[.05, 1.11]	.53	[.12, 2.29]	1.27	[.53, 3.03]
Invalidation of LGBTQ identity	.06	[.001, 1.91]	.10	[.003, 2.99]	.12	[.004, 3.39]	**.00** ^a^	[.00, 0.35]	**2.74**	[1.19, 6.32]
Microaggressions as humor	.74	[.14, 4.06]	1.12	[.30, 4.25]	.82	[.20, 3.43]	1.51	[.45, 5.02]	1.52	[.56, 4.08]
Familial microaggressions	.38	[.06, 3.11]	.38	[.06, 2.33]	.53	[.09, 3.02]	.60	[.13, 2.81]	**4.46**	[1.71, 11.63]
Threatening behaviors	.84	[.15, 4.60]	.52	[.11, 2.39]	.30	[.06, 1.51]	1.80	[.46, 7.04]	**4.58**	[1.65, 12.71]

*Note. OR,* odds ratio. *CI*, confidence interval.

Bold numbers indicate significance at *p* < .05.

All Firth logistic regression analyses controlled for the number of days participated.

^a^*OR* = .004, 95% *CI* = .0001–.35.

### Conclusion

All sexual and gender identity-based microaggression types were reported during the 14-day period of the study, except the *denial of the reality of heterosexism/transphobia*. In general, lesbian women and bisexual participants were less likely to report any microaggression type than gay participants. Concerning specific microaggression types, bisexual participants were less likely to experience *heterosexist or transphobic terminology* than gay participants. Participants assigned male at birth were less likely to experience *invalidation of LGBTQ identity* than participants assigned female at birth. Last, gender minority participants were more likely to report e*xperiences with invalidation of LGBTQ identity*, *familial microaggressions*, and *threatening behaviors*.

## Discussion

The present study aimed to examine (1) the types of sexual and gender identity-based microaggressions SGM youth report and (2) differences in these experiences by sexual and gender identity, and sex assigned at birth. To this end, data from two studies among Dutch SGM youth were used.

Across both studies, all previously identified sexual and gender identity-related microaggression types were reported by SGM youth. However, there was sizable variability in the reported frequency. Across both studies, most SGM youth reported microaggression types in which they felt that they were treated with disrespect or condemnation and in which heterosexist/transphobic language was used to degrade SGM people. Given that these findings were observed in two independent studies, using different questions with varying time frames provides strong evidence of what types of microaggressions SGM youth frequently experience. It is not surprising, however, that these types of microaggressions are reported by most SGM youth. Previous studies have shown widespread use of heterosexist and transphobic terminology in schools (Kosciw et al., 2018) and much of the (social) media messaging that youth come across includes derogatory terms and exclusionary language (Kosciw et al., 2018; Sinclair et al., 2012). Further, considering the polarization of discussions around access to affirmative healthcare, conversion therapy, access to bathrooms, participation in sports, and inclusive sexuality education, SGM youth are likely to be confronted with disapproval of their sexual or gender identity on an almost daily basis.

Interestingly, across both studies, several microaggressions were reported by less than 10% of SGM youth, for example, *denial of individual heterosexism/transphobia*, *denial of the reality of heterosexism/transphobia*, *denial of personal body privacy*, and *systemic and environmental microaggressions*. Several potential explanations may account for the low frequency of experiences with these microaggression types in our samples. First, although these forms of microaggressions may simply occur less frequently, it is also possible that they are more covert and less explicit to SGM youth, making it more difficult for these youth to describe the experiences. Second, some of the less frequently reported microaggression types were initially identified in a sample of gender minority people (Nadal et al., 2012). In the present study, a relatively small proportion of SGM youth reported a minority gender identity, which might explain why these microaggression types were reported less often. Third, the microaggression framework is partially based on the subjective experiences of minority groups—it lies in the eye of the beholder whether a situation is perceived as a microaggression (Lilienfeld, 2017). Although the subjective nature of microaggressions does not decrease the impact, some microaggression types may be reported less often than others because they were not perceived as a microaggressive event. Last, heteronormative culture is so ingrained in our society that stigma occurs at different levels, some of which may not stand out to SGM youth. For example, school practices and curricula might be rooted in heteronormativity (Steck & Perry, 2018) which is rarely seen as overtly discriminatory and may therefore be less noticeable to SGM youth, making it difficult to put these experiences into words.

We found several differences in the experience of microaggressions by sexual identity and sex assigned at birth. For sexual identity, we found that lesbian women and bisexual youth were less likely to report any microaggression type than gay youth. This difference between lesbian women and gay youth could be explained by the finding that sexual minority men, in general, experience more victimization, and perhaps also microaggressions (Pachankis et al., 2020). The difference with bisexual youth and gay men can be explained by the finding that gay men have higher levels of outness which has been associated with more victimization (Kosciw et al., 2015; Pachankis et al., 2020). This could also explain our finding that bisexual youth were less likely to report experiences in which heterosexist/transphobic language was used than gay men. Focusing on sex assigned at birth, youth assigned male at birth were less likely to report experiences in which their SGM identity was undermined or questioned than youth assigned female at birth, which was in line with expectations that cisgender women may experience daily indignities more often than cisgender men (Lewis, 2017; Swim et al., 2001). Last, we found several differences in the occurrence of microaggressions by gender identity. Gender minority youth were more likely to report experiences in which their sexual or gender identity was questioned by others, disapproval by family in a microaggressive manner, and experiences of non-physical assault. Previous studies have shown that gender minority people often face the highest levels of prejudice and stigma compared to cisgender sexual minority people (Martín-Castillo et al., 2020; Su et al., 2016). It is therefore not surprising that gender minority youth were more likely to experience situations in which their gender identity was questioned by others or experiences with non-physical assault. Further, experiences with disapproval by family in a microaggressive manner was originally identified in a sample of transgender people (Nadal et al., 2012) and therefore it could have been expected that gender minority youth would reported these experiences more often. Because the gender binary is strongly engrained in western culture and is topic of several heated societal debates, it is not surprising that gender minority youth are confronted with their gender identity being questioned by others, that they face disapproval by family, and experience non-physical assault.

Our relatively small sample sizes could have led to low statistical power to detect differences in the occurrence of microaggression types by sexual and gender identity, and sex assigned at birth in Study 2. Previous research points to differences by sexual identity (Nadal et al., 2016) and sex assigned at birth (Capodilupo et al., 2010; Nadal et al., 2016). Therefore, we should be careful to interpret our results as null findings, especially because we were unable to assess group differences for each sexual and gender identity-based microaggression type.

Taken together, Dutch SGM youth reported most of the previously formulated sexual and gender identity-based microaggression types. This is important considering that the sexual and gender identity-based microaggression types were previously deducted from theoretical work or in qualitative studies but had not yet been tested in a larger quantitative framework. Our findings are informative because experiences with microaggressions may play a role in health disparities among SGM youth. In creating safe spaces for SGM youth in schools and healthcare, we should also be wary that discomfort and disapproval of SGM youth as well as the use of heterosexist or transphobic terminology is common. Especially considering that these experiences could contribute to poorer mental health (Kaufman et al., 2017). Further, our research points to gender minority youth as an important group that experiences microaggressions most frequently.

### Limitations and Future Directions

The findings of the present study should be considered in light of some limitations. Including two independent samples of SGM youth came with at least three disadvantages. First, questions assessing sexual and gender identity-based microaggressions were phrased differently across the two studies. This could have resulted in a different interpretation of the question. For example, in Study 2, youth were explicitly asked to describe *negative* experiences related to *their* sexual orientation or gender identity, which might have resulted in youth more frequently reporting explicit microaggressions (e.g., *non-physical assault*) than youth in Study 1*.* Similarly, in Study 1 SGM youth may have reported experiences that were not directed at them, but overheard among friends or in school, explaining why *use of heterosexist/transphobic terminology* was more often reported in Study 1 than in Study 2. Further, in Study 2, participants were explicitly asked to reflect on potential experiences related to their gender identity, whereas in Study 1 they were not. This could have affected the microaggressions types reported by gender minority youth in Study 1. Last, on the first day of Study 1, youth could describe experiences that happened in the past year, whereas in Study 2 they were asked to describe experiences in the past 24 hours. This could have affected the frequency with which some microaggressions were reported.

Second, another difference between Study 1 and Study 2 is the duration of the studies. In Study 1, youth participated in eight daily diaries, whereas in Study 2, they participated in 14 diaries. Because of the shorter time frame of Study 1, it could be that some experiences with microaggressions fell outside of the study’s sampling period and were therefore missed. Similarly, the smaller sample size of Study 1 could also have resulted in fewer reported microaggressions.

Third, participants in Study 1 were sampled through advertisements on websites of LGBT community-based organizations, whereas in Study 2 participants were recruited through general advertisements on social media. These different sampling strategies could potentially explain some of the differences in demographic characteristics. For example, in Study 2 more participants identified with “emergent” sexual identity labels (e.g., queer and pansexual) and less with identity labels such as lesbian, compared to Study 1. Thus, the broader targeting of sexual minority youth in Study 2 could have resulted in targeting a more diverse population compared to the narrower targeting of people who visited websites of LGBT community-based organizations.

Besides, some more general limitations should be mentioned as well. For instance, because the reported frequency of some sexual and gender identity-based microaggressions was low, we could not assess differences by sexual identity and gender identity, and sex assigned at birth for these microaggression types. We encourage future research to quantitatively study differences by sexual identity and gender identity, and sex assigned at birth in the experience of sexual and gender identity-based microaggressions.

Further, due to relatively small SGM subgroups, we were unable to identify and compare youth with emerging sexual identities (e.g., queer or pansexual) or youth with various gender minority identities (e.g., non-binary).

Last, the majority of the microaggression literature focusses on the US and our study uniquely examines microaggressions in the Netherlands. However, both samples were not particularly diverse with regard to race/ethnicity. That is, most participants did not have a migration background. Sexual minority youth should not be viewed as a homogenous group with homogenous experiences, but rather as individuals with intersecting identities which shapes their experiences. Future research on sexual and gender identity-based microaggressions should therefore aim to study experiences of youth in more diverse samples. For the Dutch context, collecting samples of SGM youth with larger numbers of youth with a migration background from, for example, Turkey, Morocco, or Suriname could be relevant.

We outline several suggestions for future research. First, future research should focus on factors that make youth more vulnerable to the impact of microaggressions on their health and wellbeing. For example, rejection sensitivity (Downey & Feldman, 1996; Feinstein, 2020) may be a relevant construct to consider in research on the impact of microaggressions. Youth high in rejection sensitivity may perceive ambiguous events as microaggressions and learn to anxiously expect these events in the future (Baams et al., 2020), increasing the impact on their health and wellbeing.

Second, future research could expand the focus beyond microaggression types to also study heterogeneity in the occurrence of microassaults, microinsults, and microinvalidations (Sue et al., 2007) among SGM youth. For example, more overt forms of microaggressions were reported more frequently in Study 2, which might be characterized as microassaults or microinvalidations, whereas microinsults were more prevalent in Study 1. Focusing on these three forms of microaggressions could be a more parsimonious approach to study microaggressions among SGM youth.

Last, future research should consider whether all identified sexual and gender identity-based microaggression types are indeed *micro*aggressions or whether they could better be conceptualized as regular/macro aggressive events. For example, we might question whether the microaggression type *threatening behaviors*, understood as conscious non-physical assault (Nadal et al., 2011), is a microaggression or should be considered an overt form of discrimination or verbal assault. This is especially important in research on mental health, as it is currently unclear whether different types of micro-and macro-aggressions differ in their impact on mental health outcomes.

### Conclusion

This study found that SGM youth reported all previously described sexual and gender identity-based microaggressions and found that experiences in which participants felt that they were treated with disrespect or condemnation and in which heterosexist/transphobic language was used were most commonly experienced. Taken together, the present study was able to study the occurrence of microaggressions in a quantitative framework, providing a better understanding of the heterogeneity of these experiences among SGM youth.
